# Composite repair bond strength to zirconia, resin matrix CAD/CAM ceramic, and PMMA-based CAD/CAM polymer after different conditioning protocols: an in vitro investigation

**DOI:** 10.1186/s12903-026-08722-8

**Published:** 2026-05-28

**Authors:** Malin Janson, Christoph Matthias Schoppmeier, Anja Liebermann

**Affiliations:** 1 Faculty of Medicine and University Hospital Cologne, Department of Prosthetic Dentistry, University of Cologne, Cologne, Germany; 2 Faculty of Medicine and University Hospital Cologne, Polyclinic for Operative Dentistry and Periodontology, University of Cologne, Cologne, Germany; 3 Faculty of Medicine and University Hospital Cologne, Department of Prosthetic Dentistry, University of Cologne, Cologne, Germany

**Keywords:** CAD/CAM materials, Zirconia, PMMA, Repair, Universal adhesive, Shear bond strength

## Abstract

**Background:**

This in vitro investigation evaluated the effect of conditioning protocol and thermal aging on the shear bond strength (SBS) of composite repair to zirconia, a resin matrix CAD/CAM ceramic, and a PMMA-based CAD/CAM polymer.

**Methods:**

A total of 120 specimens were fabricated from zirconia (Katana Zirconia HT), a resin matrix CAD/CAM ceramic (Grandio Disc), and a PMMA-based CAD/CAM polymer (Structur CAD). All specimens were standardized by airborne-particle abrasion using 50 μm Al₂O₃ (1 bar, 10 s). For zirconia and Grandio Disc, two conditioning protocols were applied: universal adhesive alone (Clearfil Universal Bond Quick) and ceramic primer combined with universal adhesive (Clearfil Ceramic Primer Plus). Structur CAD was treated with the universal adhesive only, in accordance with the manufacturer’s recommendation. Composite (Clearfil Majesty ES-2 Universal) was applied, and SBS (MPa) was measured after 24-hour storage and after thermocycling (5000 cycles, 5–55 °C). Failure modes were evaluated at 40× magnification. Statistical analysis was performed using a three-factor ANOVA with Type II sums of squares computed by model comparison, allowing estimation of the effects considered estimable within the incomplete factorial design. Post hoc pairwise comparisons were performed with Bonferroni correction (α = 0.05).

**Results:**

Shear bond strength was significantly affected by material (*p* < 0.001) and aging (*p* < 0.001). For the subset of materials in which both conditioning protocols were available (zirconia and resin matrix CAD/CAM ceramic), conditioning protocol also showed a significant effect (*p* = 0.013), with a significant material × conditioning interaction (*p* = 0.042). A significant material × aging interaction was observed (*p* < 0.001). For Grandio Disc, ceramic primer application did not significantly alter SBS. In zirconia, ceramic primer increased SBS prior to aging (*p* = 0.004), whereas this increase in SBS was no longer observed after thermocycling (*p* = 0.102). Thermocycling reduced SBS across all groups, most pronouncedly in Structur CAD. Failure modes were predominantly adhesive and mixed.

**Conclusion:**

Repair bond strength is material-dependent and reduced by thermal aging. Additional ceramic primer application did not improve bond strength for the resin matrix CAD/CAM ceramic and showed no sustained benefit for zirconia after aging. The PMMA-based CAD/CAM polymer exhibited the greatest aging-related reduction, suggesting that material-specific conditioning strategies warrant further investigation.

## Background

CAD/CAM restorative materials encompass several material classes, including glass-ceramics, zirconia, resin matrix CAD/CAM ceramics, and PMMA-based polymers, and have become integral to contemporary prosthodontics because industrial fabrication enables high dimensional accuracy, standardized material quality, and efficient digital workflows [1]. However, despite continuous material development, clinical complications such as chipping, fractures, wear, and marginal defects remain frequent reasons for intervention [2].

While zirconia and silicate ceramics mainly exhibit chipping or veneer-related defects, these can often be repaired using adhesive systems and composite materials [3]. Resin matrix and polymer-based CAD/CAM materials are more frequently affected by abrasion, microcracks, or material fatigue. Many of these defects are localized and may be treated using minimally invasive intraoral composite repair, thereby preserving sound tooth structure and reducing treatment costs [4, 5]. The success of such repairs depends largely on durable adhesion between the repair composite and the aged restorative substrate. This is particularly challenging in CAD/CAM materials, because their chemical composition and microstructure differ substantially and directly influence surface reactivity and bonding behavior [1]. Among the broad range of CAD/CAM restorative materials, the present study focused on three clinically relevant material classes representing distinct bonding substrates: polycrystalline oxide ceramics such as zirconia, resin matrix CAD/CAM ceramics with dispersed inorganic fillers, and PMMA-based CAD/CAM polymers.

For zirconia, adhesive bonding principles are relatively well established, particularly the use of airborne-particle abrasion combined with phosphate monomers such as 10-MDP. Owing to its polycrystalline, glass-free structure, zirconia cannot be conditioned by conventional silane-based protocols alone. Instead, a combined mechanical–chemical approach based on airborne-particle abrasion and functional phosphate monomers such as 10-MDP is widely recommended and has been shown to provide reliable and hydrolytically stable bonding [5, 6].

In contrast, consensus regarding polymer-based CAD/CAM materials is more limited. Resin matrix CAD/CAM ceramics differ fundamentally from zirconia because they consist of an organic matrix with dispersed inorganic filler particles, which may permit both micromechanical interlocking and chemical coupling via silane [7]. Previous studies on related resin matrix CAD/CAM materials indicate material-dependent effects of surface pretreatment and silanization, yet direct evidence for Grandio Disc, particularly under aging conditions, remains limited. Because most studies focus on individual materials rather than cross-material comparisons, the transferability of findings between CAD/CAM materials remains unclear [7, 8].

In addition, the hydrolytic stability of silane components incorporated in universal adhesives has been questioned, suggesting that separate silanization may be advantageous under certain conditions [9].

PMMA-based CAD/CAM polymers such as Structur CAD are industrially polymerized and exhibit a dense, highly cross-linked polymer structure with limited reactive surface groups [9, 10]. In contrast to resin matrix CAD/CAM ceramics, they do not contain an inorganic filler phase, so silane-mediated coupling is not expected to contribute to adhesion [9, 11, 12]. Previous findings from related PMMA materials suggest that bonding may depend mainly on micromechanical retention and, potentially, on MMA-based chemical surface activation [9]. However, for Structur CAD itself, independent data on repair bond strength remain scarce.

Despite increasing use of these materials, comparative evidence on repair bond strength across materially distinct CAD/CAM substrates under standardized and aged conditions remains limited, and direct cross-material comparisons within a unified experimental design are lacking. Because conditioning strategies are substrate-dependent and vary according to material composition, the repair protocol used in the present study was deliberately selected based on manufacturer recommendations for the investigated materials. Airborne-particle abrasion, an established conditioning approach particularly for zirconia-based substrates, served as the common experimental basis for standardized comparison among the investigated substrates. Therefore, the aim of this in vitro study was to compare the repair bond strength of resin composite to zirconia, a resin matrix CAD/CAM material, and a PMMA-based CAD/CAM polymer after standardized airborne-particle abrasion and adhesive conditioning, before and after thermocycling. For zirconia and Grandio Disc, the effect of a silane-containing ceramic primer in combination with a universal adhesive was compared with the use of the universal adhesive alone. For Structur CAD, repair was performed using the universal adhesive alone in accordance with the manufacturer’s recommendation. The null hypotheses were that (1) for zirconia and the resin matrix CAD/CAM material, the additional use of ceramic primer would not affect repair bond strength compared with the universal adhesive alone, and (2) thermal aging would not influence repair bond strength of the investigated materials.

## Methods

### Sample size calculation

Sample size calculation was performed a priori using G*Power software (version 3.1.9.6; Franz Faul, Kiel University, Kiel, Germany). As the primary outcome of interest was the pairwise comparison of shear bond strength between experimental groups, the calculation was based on a two-tailed independent-samples t-test assuming a large effect size (Cohen’s d = 1.2), a statistical power (1-β) of 0.80, and a significance level of α = 0.05. The assumed effect size was derived from previous investigations reporting comparable magnitudes of difference in shear bond strength between conditioning protocols for CAD/CAM materials. Based on this calculation, a minimum of 12 specimens per group was required. Accordingly, a total of 120 specimens were prepared and allocated to the ten experimental subgroups.

A three-factor ANOVA was subsequently applied to analyze main and interaction effects within the experimental design.

### Specimen preparation

Three CAD/CAM materials were included in this study: a polycrystalline zirconia (KAT; Katana Zirconia HT, Kuraray Noritake, Okayama, Japan), a highly filled resin matrix CAD/CAM ceramic (GRD; Grandio Disc, VOCO GmbH, Cuxhaven, Germany), and a PMMA-based CAD/CAM polymer (STC; Structur CAD, VOCO GmbH, Cuxhaven, Germany).

Rectangular specimens measuring 10 × 10 × 2 mm were fabricated from each material. Specimens of Grandio Disc and Structur CAD were milled using a CNC milling unit (Ceramill Motion 2, Amann Girrbach, Koblach, Austria). Zirconia specimens were additionally sintered in accordance with the manufacturer’s instructions in a sintering furnace (Ceramill Therm 3, Amann Girrbach, Koblach, Austria).

### Surface preparation

All specimens underwent standardized surface preparation under water cooling using silicon carbide abrasive papers ranging from P600 to P1200 (SiC Foil, Struers, Ballerup, Denmark). Subsequently, the specimens were ultrasonically cleaned in 96% isopropanol for 3 min and air-dried for 10 s. Afterwards, they were ultrasonically cleaned with distilled water for 10 min and dried again with air.

The final specimen thickness was digitally verified (Alpha Tools, Franklin, USA). To ensure comparable baseline conditions, all specimens were subjected to identical mechanical surface conditioning by airborne-particle abrasion using 50 μm aluminum oxide at 1 bar pressure for 10 s from a distance of 10 mm [5]. A comparatively low abrasion pressure was deliberately selected to avoid excessive surface damage in the polymer-based materials while maintaining a uniform protocol across all substrates. While higher pressures can be used for zirconia to achieve optimal surface roughening, lower-pressure protocols have also been described in the literature [5].

### Experimental groups and bonding procedures

**Fig. 1:**
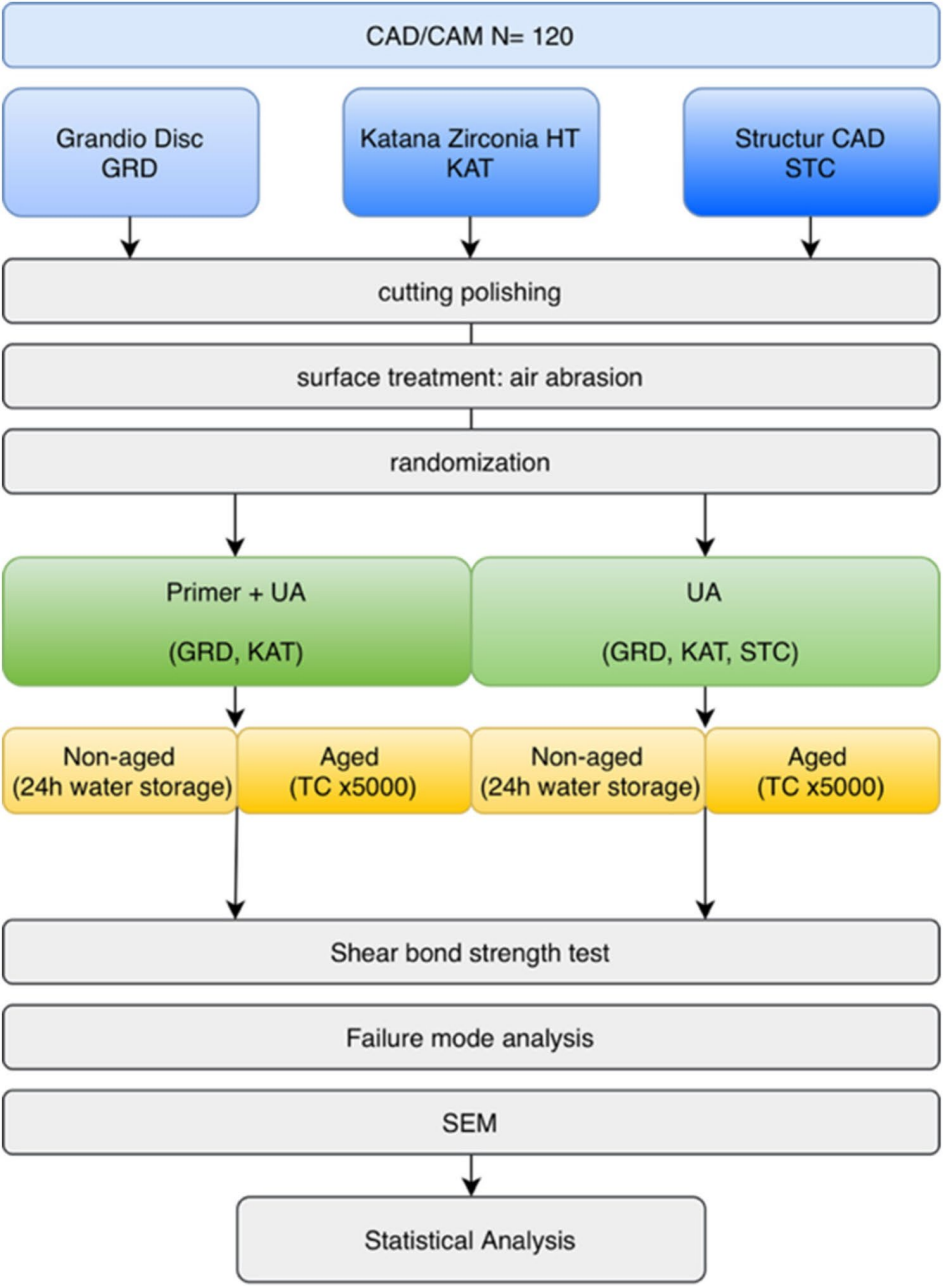
Study flowchart

Specimens were randomly allocated to the respective material and treatment groups. Randomization was performed after specimen preparation and before application of the respective conditioning protocol. The allocation of specimens and the workflow of the repair protocol are illustrated in Fig. 1, while the materials used, manufacturer information, and application protocols are summarized in Table 1.

**Table 1:** Brand names, manufacturers, batch numbers, chemical composition and applications of materials used

Brand/Manufacturer	Abbreviation	Batch number	Chemical Composition	Application
Grandio Disc Voco Dental Germany	GRD	2,424,673	Nanohybrid composite 86.0 wt% inorganic fillers	
Structur CAD Voco Dental Germany	STC	2,426,768	Polymer matrix (methacrylate), 27.0 wt% inorganic fillers	
Katana Zirconia HT / Kuraray Noritake Dental Inc, Okayama, Japan	KAT	EKQQH	Zirconium dioxide, ZrO2 (80.0–95.0%), yttrium oxide, Y2O3 (3.0–15.0%), titanium dioxide (0–10%), pigments.	-
Composite Clearfil Majesty ES-2 Universal Kuraray Noritake Dental Inc, Okayama, Japan	CM	430,020	Silanated barium glass filler, pre-polymerized organic filler (0.0–40.0), Bisphenol A diglycidylmethacrylate, Hydrophobic aromatic dimethacrylate, Silanated colloidal silica/Hydrophobic aliphatic dimethacrylate, dl-Camphorquin	Applied and light cured in 2 × 1 mm increments
Korox 50, Bego GmbH, Bremen, Deutschland/ Dento-Prep; Ronvig, Daugaard, Dänemark			50 μm Al_2_O_3_-Particles	Pressure of 1 bar/0.1 MPa for 10 s at a distance of 10 mm
Clearfil Ceramic Primer Plus + Clearfil Universal Bond Quick / Kuraray Noritake Dental Inc., Okayama, Japan	CUBQ	230,091 220,393	3-Trimethoxysilylpropylmethacrylat 10-Methacryloyloxydecyl-Dihydrogenphosphat, Ethanol MDP, Bis-GMA, HEMA, hydrophilic aliphatic dimethacrylate, colloidal silica, silane coupling agent, dl-camphor quinone, ethanol, water, Sodium fluoride	Applied Ceramic Primer, evaporated for 20 s, air died. Applied and massaged in for 20 s, air dried for 10 s, light cured for 10 s.

*Abbreviations*: *MDP* 10-Methacryloyloxydecyl-Dihydrogenphosphat, *Bis-GMA* Bisphenol-A diglycidylmethacrylate, HEMA 2-Hydroxyethylmethacrylat

For Grandio Disc and Katana Zirconia HT, two subgroups were defined:

A. Application of a universal adhesive (Clearfil Universal Bond Quick, Kuraray Noritake, Okayama, Japan) without prior ceramic primer application.
B. Application of a silane-containing ceramic primer (Clearfil Ceramic Primer Plus, Kuraray Noritake, Okayama, Japan) followed by application of the universal adhesive (Clearfil Universal Bond Quick).

For Structur CAD, a separate group was defined:

C.  Application of the universal adhesive (Clearfil Universal Bond Quick) without additional chemical pretreatment, in accordance with the manufacturer’s recommendations for PMMA-based CAD/CAM polymers.

All materials were applied strictly according to the manufacturers’ instructions. For the ceramic primer subgroup, Clearfil Ceramic Primer Plus was applied to the air-abraded surface using a microbrush for 20 s and gently air-dried for 10 s. Subsequently, Clearfil Universal Bond Quick was applied with a microbrush for 20 s, air-thinned for 10 s to evaporate the bonding solvent, and light-cured using LED with 1200 mW/cm2 intensity (Bluephase Style, Ivoclar Vivadent, Schaan, Liechtenstein).

In the universal-adhesive-only groups, Clearfil Universal Bond Quick was applied using a microbrush for 20 s, gently air-dried for 10 s and light-cured using LED with 1200 mW/cm2 intensity (Bluephase Style, Ivoclar Vivadent, Schaan, Liechtenstein).

### Composite build-up

Composite cylinders were subsequently fabricated on the treated specimen surfaces. Each specimen was positioned in a silicone mold to ensure precise centering of a standardized Teflon mold (5 mm diameter × 2 mm height).

The composite material (Clearfil Majesty ES-2 Universal, Kuraray Noritake, Okayama, Japan) was applied in two layers of 1 mm each. Each layer was light-cured for 20 s using an LED curing unit (Bluephase Style, Ivoclar Vivadent, Ellwangen, Germany; 1200 mW/cm²) at a distance of approximately 1 mm. After removal of the Teflon mold, an additional 20-second polymerization cycle was performed.

The chosen bonded area and composite cylinder geometry were standardized for all groups to ensure reproducible specimen preparation and testing conditions.

### Aging procedure

All specimens were initially stored in distilled water at 37 °C for 24 h. Subsequently, half of the specimens from each group were subjected to thermal aging consisting of 5000 thermocycles between 5 °C and 55 °C (dwell time: 30 s; transfer time: 5 s) using a thermocycling device (RC 20 CS Lauda, Lauda-Königshofen, Germany).

Thermocycling was selected as an established in vitro aging method to simulate hydrothermal stress at the bonded interface. Mechanical fatigue loading was not included in the present study and was therefore considered beyond the scope of the experimental design.

### Shear bond strength testing

Prior to mechanical testing, all specimens were ultrasonically cleaned in distilled water for 5 min and dried with oil-free compressed air.

Shear bond strength was measured using a universal testing machine (zwickiLine Z0.5 TN, Zwick Roell, Ulm, Germany) at a crosshead speed of 0.5 mm/min. The maximum load at failure (N) was divided by the bonded surface area (19.63 mm²) to calculate shear bond strength values expressed in MPa.

During testing, the bonded interface was aligned parallel to the loading direction. Shear force was applied using a knife-edge indenter positioned as close as possible to the interface between the composite cylinder and the substrate.

Specimen positioning during testing was performed by the same operator for all groups to ensure standardized alignment.

### Failure mode analysis

Failure modes were analyzed using a digital microscope (VHX-5000, Keyence, Osaka, Japan) at 40× magnification and categorized as adhesive, cohesive, or mixed failures. Two independent examiners performed the evaluation, and discrepancies were resolved by consensus.

Additionally, one representative specimen from each group was prepared for scanning electron microscopy (SEM) analysis (Prisma ESEM, Thermo Fisher Scientific, Waltham, USA). Specimens were sputter-coated with gold–palladium (Q150T Plus, Quorum Technologies, East Sussex, UK) and examined at 1000× magnification. SEM evaluation was performed as a qualitative, descriptive assessment focusing on interfacial and fracture-related features rather than general surface topography. The selected magnification enabled visualization of microstructural characteristics such as crack propagation patterns, material discontinuities, and interfacial integrity, which are not sufficiently resolved at lower magnifications. Accordingly, SEM imaging was used to provide representative, mechanistic insight into failure modes before and after aging and was not intended for quantitative analysis.

### Statistical analysis

Statistical analyses were performed using R (R Foundation for Statistical Computing, Vienna, Austria). Prior to analysis, normality of the residuals was assessed using the Shapiro–Wilk test, and homogeneity of variances was verified using Levene’s test. To evaluate the effects of material, conditioning protocol, and thermal aging on shear bond strength, a three-factor analysis of variance (ANOVA; Material × Conditioning × Aging) was performed. Because no primer-treated subgroup was included for Structur CAD, the experimental design was structurally incomplete with respect to the conditioning factor. To accommodate this, Type II sums of squares were computed by model comparison using the *car* package, which provides valid estimates restricted to the set of estimable effects. Conditioning-related interaction terms were evaluated only for the subset of materials for which both conditioning levels were available (KAT and GRD). Post hoc pairwise comparisons were conducted on estimated marginal means using the *emmeans* package with Bonferroni correction. Effect sizes were quantified using partial eta squared (η²) and reported together with F- and p-values. Effects involving the conditioning protocol were interpreted with reference to the KAT and GRD subgroups only. The level of statistical significance was set at α = 0.05.

## Results

### Shear bond strength

The mean shear bond strength (SBS) values and standard deviations for all groups are presented in Table 2. Under non-aged conditions and universal-adhesive-only repair, GRD (29.42 ± 2.61 MPa) and STC (29.58 ± 4.01 MPa) showed comparable SBS values, whereas KAT showed lower values (16.40 ± 3.13 MPa). When ceramic primer was additionally applied, GRD showed significantly higher SBS values than KAT both before and after thermal aging. After thermocycling, SBS values were lower in all groups, and significant differences were observed among the investigated materials. Because Structur CAD was evaluated under a single conditioning protocol, effects involving the conditioning factor, including material × conditioning interactions, were interpreted only for zirconia and Grandio Disc.

**Table 2:** Shear bond strength (MPa, mean ± SD) of composite repair to different CAD/CAM materials before and after thermal aging

Group	Non-aged MPa	Aged MPa
KAT UA	16.40 $\:\pm\:$ 3.13^a^	14.02 $\:\pm\:\:$3.30^a^*
KAT UA+Primer	20.08 $\:\pm\:\:$3.45^b^	16.08 $\:\pm\:$ 1.00^a^*
GRD UA	29.42$\:\:\pm\:$ 2.61^c^	20.92$\:\:\pm\:$ 2.02^c^*
GRD UA+Primer	28.67 $\:\pm\:$ 2.64^c^	22.25 $\:\pm\:$ 2.63^c^*
STC UA	29.58 $\:\pm\:$ 4.01^c^	17.42 $\:\pm\:$ 4.44^b^*
Total	24.83 $\:\pm\:$ 6.37	18.14 $\:\pm\:\:4$0.17

Different superscript letters indicate statistically significant differences between groups within the same aging condition (*p* < 0.05, Bonferroni-adjusted post hoc comparisons). Groups sharing the same superscript letter do not differ significantly. The symbol * indicates a significant difference between non-aged and aged specimens within the same group (*p* < 0.05)

*Abbreviations*: *KAT* Katana Zirconia HT, *GRD* Grandio Disc, *STC* Structur CAD, *UA* Universal Adhesive

### Model assumptions

Prior to analysis, model assumptions were examined. The Shapiro–Wilk test of residuals indicated a slight deviation from normality (W = 0.976, *p* = 0.031), whereas Levene’s test showed no significant violation of homogeneity of variances (*p* = 0.063). Given the robustness of ANOVA against minor deviations from normality with balanced cell sizes ≥ 10, parametric analysis was considered appropriate.

### Effects of material, conditioning protocol, and aging

The three-factor ANOVA revealed significant main effects of material (F(2, 110) = 105.04, *p* < 0.001, partial η² = 0.656), conditioning protocol (F(1, 110) = 6.38, *p* = 0.013, partial η² = 0.055), and thermal aging (F(1, 110) = 142.74, *p* < 0.001, partial η² = 0.565). Significant two-way interactions were observed between material and conditioning protocol (F(1, 110) = 4.25, *p* = 0.042, partial η² = 0.037) and between material and aging (F(2, 110) = 15.42, *p* < 0.001, partial η² = 0.219), indicating that the effect of the ceramic primer differed between materials and that the magnitude of the aging-related SBS reduction was material-dependent. No significant interaction was found between conditioning protocol and aging (F(1, 110) = 0.03, *p* = 0.854, partial η² < 0.001), and no significant three-way interaction was detected (F(1, 110) = 2.19, *p* = 0.142, partial η² = 0.019). The complete ANOVA results are presented in Table 3.

**Table 3:** Three-way ANOVA results for shear bond strength (SBS) showing the effects of material, repair strategy, thermal aging, and their interactions

	df		F		*p*-value	partial η²
Material	2		105.04		< 0.001	0.656
Repair strategy (Bonding)	1		6.38		0.013	0.055
Ageing	1		142.74		< 0.001	0.565
Material × Repair strategy	1		4.25		0.042	0.037
Material × Ageing	2		15.42		< 0.001	0.219
Repair strategy × Ageing	1		0.03		0.854	0.000
Material × Repair strategy × Ageing	1		2.19		0.142	0.019

### Material-specific effect of additional ceramic primer

For GRD, no statistically significant difference in SBS was observed between the universal adhesive alone and the combination of ceramic primer and universal adhesive, either before thermal aging (*p* = 0.551) or after thermocycling (*p* = 0.290). For KAT, the additional application of ceramic primer resulted in significantly higher SBS values before thermal aging, increasing from 16.40 ± 3.13 MPa to 20.08 ± 3.45 MPa (*p* = 0.004). After thermocycling, this difference was no longer statistically significant (14.02 ± 3.30 MPa vs. 16.08 ± 1.00 MPa, *p* = 0.102).

### Effect of thermal aging

Thermocycling resulted in lower SBS values in all investigated groups. The greatest aging-related reduction was observed for STC, with SBS decreasing from 29.58 ± 4.01 MPa to 17.42 ± 4.44 MPa after thermocycling, followed by KAT and GRD. The significant material × aging interaction confirmed that the extent of aging-related degradation was material-dependent, whereas no significant interaction between conditioning protocol and aging was detected.

### Failure mode analysis

**Fig. 2:**
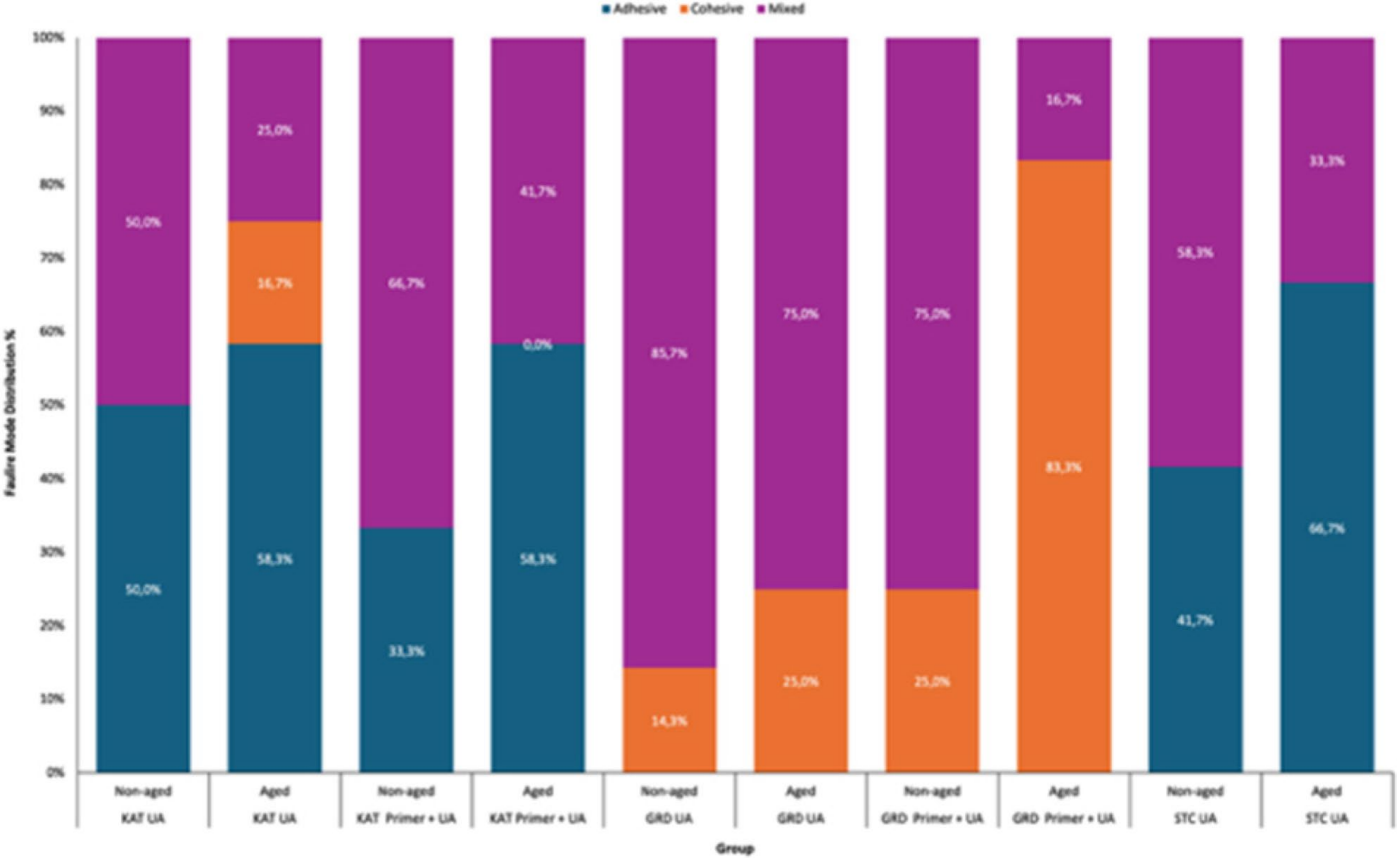
Distribution of failure modes after shear bond strength testing for all experimental groups after 24h water storage (non-aged) and 30 days water storage and thermally aged conditions

The distribution of failure modes is shown in Fig. 2. In the KAT groups, adhesive and mixed failures were observed both before and after thermocycling, whereas cohesive failures occurred only rarely. Additional ceramic primer application did not result in a marked change in failure mode distribution. In the GRD groups, mixed failures were observed under non-aged conditions. After thermocycling, a higher proportion of cohesive failures was observed, particularly in the primer-treated subgroup. In the STC group, adhesive and mixed failures were observed before aging, whereas after thermocycling the proportion of adhesive failures increased.

### SEM observations

**Fig. 3:**
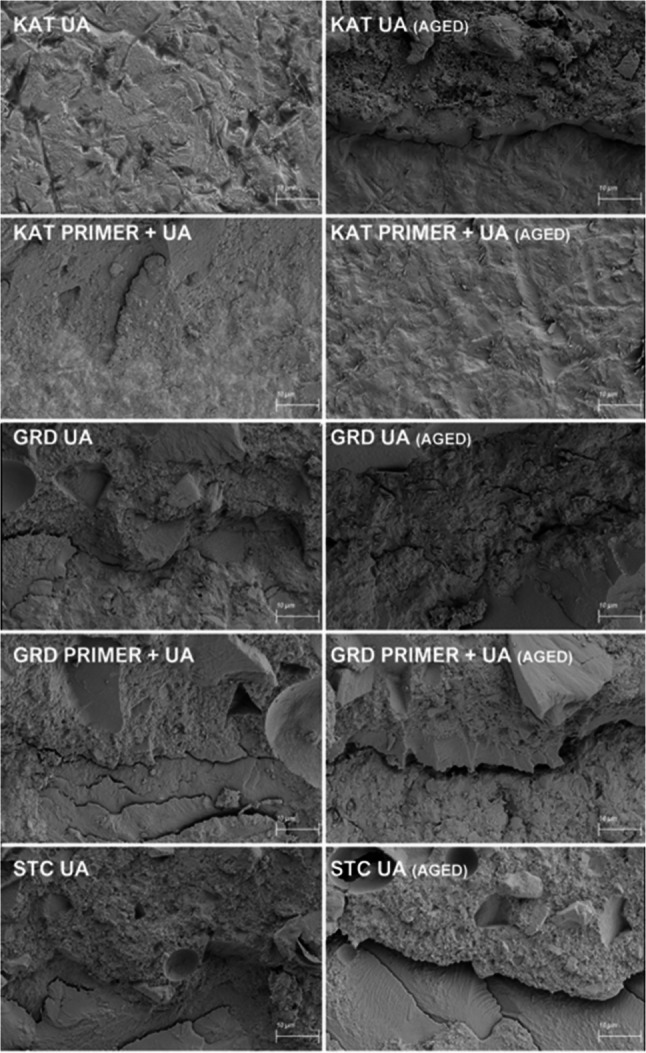
Representative scanning electron microscope (SEM) images of the adhesive interfaces for the investigated CAD/CAM materials under non-aged and thermally aged conditions, obtained at 1000× magnification

Representative SEM images of non-aged and aged specimens are presented in Fig. 3. Zirconia specimens exhibited predominantly rough and granular fracture morphologies. In groups treated without ceramic primer, fracture surfaces appeared relatively homogeneous and showed limited composite remnants. In contrast, primer-treated zirconia specimens displayed more heterogeneous surface patterns with areas showing composite remnants. The polymer-based materials showed variable morphologic features. GRD exhibited a comparatively compact fracture structure, whereas STC showed a coarser, step-like fracture pattern with irregular surface topography. In aged specimens, discontinuities at the adhesive interface were observed more frequently. Interfacial gaps and irregular transitions between substrate and repair composite were observed. The adhesive layer showed variable thickness and inconsistent structural continuity.

## Discussion

The results led to the rejection of both null hypotheses. Material type, repair strategy, and thermal aging significantly affected repair bond strength. However, statistical significance should be distinguished from clinical relevance, particularly because the effect size of the conditioning protocol was smaller than that of material type and aging. Therefore, the conditioning-related findings should be interpreted primarily as material-specific experimental effects rather than as evidence of clinically meaningful superiority of one protocol across all substrates.

The significant interaction between material and repair strategy indicates that the effectiveness of surface conditioning depends on the substrate. In contrast, no significant interaction between repair strategy and aging was observed, indicating that the effect of aging was not significantly modified by the repair strategy. Furthermore, the absence of a significant three-way interaction indicates that no complex combined interaction among the investigated factors was present. The results revealed pronounced differences in bond strength among the investigated materials. Under non-aged conditions and without additional chemical pretreatment, GRD and STC exhibited significantly higher SBS values than KAT. After thermocycling, all three materials differed significantly from each other. These differences may be related to the structural and chemical characteristics of the respective substrate materials.

Zirconia is a polycrystalline, glass-free oxide ceramic characterized by a highly crystalline microstructure and the absence of a silicate phase. The absence of a silicate phase limits classical silane-mediated bonding mechanisms [13]. Adhesion to zirconia therefore is generally attributed to the chemical interaction of functional phosphate monomers, particularly 10-MDP, with the zirconia surface [14]. This interaction is considered to result in the formation of stable Zr-O-P bonds that exhibit comparatively high hydrolytic stability [14]. In addition, factors such as grain size, sintering density, and surface roughness after airborne-particle abrasion may influence the degree of micromechanical retention [5, 13]. Due to its high elastic modulus and negligible water absorption, zirconia remains structurally stable under thermal stress, which may contribute to the comparatively low aging sensitivity observed in the present study [15].

In the present study, KAT exhibited lower bond strength values compared with the other materials. The repair strategy significantly influenced SBS prior to thermal aging, with additional ceramic primer application resulting in higher initial bond strength values. However, this advantage was no longer observed after thermocycling, suggesting that the effect may be limited to short-term interfacial interactions. Because silane does not exhibit direct chemical affinity to zirconia, stable siloxane bonds cannot be formed [16, 17], and the initial improvement may therefore be associated with enhanced surface wetting or the formation of a transient interfacial layer.

The use of a uniform low-pressure airborne-particle abrasion protocol, while beneficial for intergroup comparability, does not fully reflect material-specific clinical recommendations. In particular, zirconia can be treated with higher abrasion pressures to enhance surface roughening [5]. Therefore, the present approach may have resulted in less pronounced surface modification and should be interpreted as a conservative estimate rather than an optimized clinical protocol. The interaction between 10-MDP and zirconia may contribute to the comparatively stable bond strength values observed after thermocycling despite lower initial SBS values. The minimal water uptake and high elastic modulus of zirconia may further contribute to the stability of the adhesive interface [5, 18].

In contrast to zirconia, GRD exhibited the highest absolute SBS values among the investigated materials both before and after thermal aging. However, no significant influence of additional primer application was observed.

Resin matrix CAD/CAM ceramics such as GRD consist of a highly cross-linked polymer matrix containing a large proportion of silicate or ceramic filler particles and are polymerized under elevated temperature and pressure during industrial manufacturing, resulting in high conversion rates, low residual monomer content, and a relatively homogeneous microstructure [19, 20]. This manufacturing process also leads to reduced water absorption compared with directly placed composite materials [19]. Exposed filler surfaces enable potential chemical coupling via silane, whereas the polymer matrix facilitates micromechanical retention following airborne-particle abrasion [7, 8]. In addition, the lower elastic modulus of the composite compared with zirconia may partially compensate for interfacial stresses generated during thermal loading.

In the present study, the absence of a significant benefit from additional silanization may be related to the presence of silane within the universal adhesive, which may already provide sufficient chemical interaction with exposed filler surfaces. Nevertheless, the stability of silane incorporated in universal adhesives has been questioned in previous literature, and additional silanization may still be advantageous under certain conditions [18]. The moderate reduction in SBS values after thermocycling is consistent with the assumption that the adhesive interface between GRD and the repair composite remains comparatively stable under hydrothermal conditions.

In contrast, PMMA-based CAD/CAM polymers consist almost entirely of a polymeric matrix without an inorganic filler phase. Industrial prepolymerization results in a dense and highly cross-linked structure with a limited number of reactive methacrylate groups at the surface [10]. Because a silicate phase is absent, silane-mediated coupling cannot contribute to adhesion. Bonding is therefore generally assumed to rely primarily on micromechanical retention and, when applied, on monomer-mediated surface activation using MMA, which may promote interdiffusion between the substrate and the repair composite [20]. In addition, PMMA exhibits higher water uptake and greater susceptibility to plastic deformation under thermal loading than zirconia, which may contribute to increased aging sensitivity.

In the present study, STC showed bond strength values comparable to GRD before thermocycling but exhibited the greatest reduction after aging and the lowest values among all materials in the aged condition. This pronounced decrease suggests limited hydrolytic stability of the repair interface. Because no MMA-based pretreatment was included, the observed bonding performance may have been governed primarily by micromechanical retention and the applied adhesive system. Under thermal aging, water sorption, plasticization, and dimensional changes may further contribute to interfacial stress and hydrolytic degradation of the bonded interface [21]. These findings indicate that, for PMMA-based CAD/CAM polymers, not only the initial bond strength but particularly the durability of the repair interface is critical. Since only one repair protocol was tested, no direct conclusions can be drawn regarding the effectiveness of alternative PMMA-specific conditioning strategies.

The clinical relevance of bond strength values remains debated. The DIN EN ISO 10,477 standard defines a minimum bond strength of 5 MPa for polymer-based restorative materials, provided that at least 80% of specimens reach this threshold [22]. However, this requirement primarily refers to polymer-based materials and is not specifically intended for ceramic bonding systems. For ceramic adhesive systems, higher bond strength ranges exceeding 10 MPa are often considered desirable, and values between approximately 15 and 20 MPa are sometimes discussed as favorable for durable adhesive bonding [23]. In the present study, the non-aged SBS values of GRD and STC exceeded these thresholds, whereas KAT remained within lower value ranges that have been discussed as potentially clinically relevant. After thermal aging, the SBS values of STC approached the lower threshold, indicating greater aging sensitivity. These threshold values should be interpreted with caution, as they cannot be directly extrapolated to clinical performance because intraoral conditions involve additional degradation mechanisms. Although statistically significant differences were detected, not all differences necessarily indicate clinically relevant superiority. Differences of a few MPa should be interpreted cautiously, particularly because SBS testing does not directly reproduce intraoral loading conditions. In contrast, the pronounced aging-related reduction observed for STC is more likely to be clinically relevant, as it shifted the material toward lower bond strength ranges after thermocycling.

Thermocycling resulted in a significant reduction in SBS values across all groups, confirming the degrading influence of hydrothermal stress on adhesive interfaces. The significant interaction between material and aging indicates that the magnitude of this degradation depends on the substrate. Thermal aging simulates repeated temperature fluctuations in combination with water exposure. Several mechanisms have been proposed to contribute to interfacial degradation, including water sorption and plasticization of polymer phases, hydrolytic cleavage of ester bonds within the resin matrix, and stress development due to mismatched thermal expansion coefficients between substrate, adhesive layer, and repair composite. Polymer-based materials such as GRD and particularly STC exhibit greater water uptake than oxide ceramics. While industrially polymerized CAD/CAM composites such as GRD demonstrate reduced water absorption due to their high cross-link density, PMMA-based materials may undergo more pronounced plasticization and dimensional changes during aging. In contrast, zirconia exhibits negligible water uptake and maintains structural stability because of its high elastic modulus [24].

The fracture morphology of the non-aged specimens was consistent with the SBS results. Zirconia specimens without ceramic primer predominantly exhibited homogeneous granular surfaces with minimal composite remnants. In contrast, primer-treated groups showed more heterogeneous fracture patterns with composite remnants, consistent with mixed failure characteristics and improved interfacial interaction. Similarly, composite-based materials demonstrated differences depending on surface pretreatment. Primer-treated specimens revealed morphological features with increased composite remnants, whereas non-treated groups showed predominantly interfacial characteristics. As all specimens were stored in water for 24 h only, hydrolytic degradation can be considered negligible. Therefore, the observed differences may be related to the applied surface conditioning rather than aging effects. These morphological observations support the mechanical findings, as groups with higher SBS values generally showed more composite remnants and mixed failure characteristics, whereas groups with lower SBS values exhibited predominantly interfacial fracture patterns. Although SEM was qualitative and based on representative specimens only, the observed fracture patterns were consistent with the SBS trends.

Several limitations of this study should be considered. Only a single universal adhesive system was investigated, and MMA-based primers were not evaluated. In addition, the factorial design was structurally incomplete because the PMMA-based material was tested only with the manufacturer-recommended universal adhesive protocol. Therefore, conditioning-related effects and interactions should only be interpreted for zirconia and the resin matrix CAD/CAM material, for which both conditioning levels were available. Furthermore, the sample size was calculated to detect between-group differences and was not specifically powered to assess higher-order interaction effects; thus, interaction effects should be interpreted with caution. In addition, the aging protocol consisted solely of thermocycling without mechanical loading, which may limit the simulation of clinical intraoral conditions. Surface roughness following airborne-particle abrasion was not quantitatively assessed, precluding direct correlation with bond strength values. Finally, shear bond strength testing does not represent a purely interfacial property, as stress distribution may be influenced by specimen geometry and loading configuration. Therefore, absolute bond strength values should be interpreted with caution. Only one repair protocol was evaluated for the PMMA-based material.

## Conclusion

Repair bond strength to CAD/CAM materials is material-dependent and significantly reduced by thermocycling. For zirconia, the tested airborne-particle abrasion and universal adhesive protocol showed a lower aging-related reduction compared with the PMMA-based CAD/CAM polymer, while additional ceramic primer application did not provide a sustained benefit. For the resin matrix CAD/CAM ceramic, additional silanization did not improve repair bond strength when used in combination with the tested universal adhesive. The PMMA-based CAD/CAM polymer exhibited the greatest aging-related reduction in bond strength, indicating that material-specific chemical activation strategies may be required to achieve durable repair. Overall, the investigated materials exhibited distinct bonding behavior, particularly after aging, emphasizing the need for material-specific repair strategies.

## Data Availability

Data will be available from the corresponding author on request.

## Abbreviations

GRD: Grandio Disc
KAT: Katana Zirconia HT
STC: Structur CAD
UA: Universal Adhesive
